# Supplementary material for: Deleterious neurological impact of diagnostic delay in immune-mediated thrombotic thrombocytopenic purpura
Source: PLoS One. 2021 Nov 19;16(11):e0260196. doi: 10.1371/journal.pone.0260196 (PMC8604371; doi:10.1371/journal.pone.0260196)
Supplement: Attachment — Submitted filename: Response to Reviewers.docx [file pone.0260196.s002.docx]

**Reviewer #1** In this manuscript, the authors investigated the effects of diagnostic delay on neurological in patients with iTTP. Based on their results, Renaud et al. concluded that diagnostic delay had a significant impact on neurological outcome. Although the results of this study add a little new information to current literatures, the retrospective nature and small number of cases make it difficult to draw a definite conclusion that “diagnostic delay has an impact on neurological outcomes in iTTP”. My comments:

**1. Why the cutoff value of 24h was used to group classification? Please add the number of patients in group 1 and group 2.**

In our county (44, Loire Atlantique, 6 880 km², 1,426 million inhabitants), access to plasma exchange (PE) is always possible within 24hours. If PE is not quickly available (night), our practice is to initiate plasma infusions. Accordingly, no timely diagnosed patients were found to receive plasma therapy more than 24h after admission. We chose this 24h cut-off because we considered that it best reflected misdiagnosis. A shorter cut-off would capture both logistical issues and misdiagnosis, whereas a longer cut-off wouldn’t be in accordance with expert recommendation regarding TTP management. Another reason was the distribution of data (Figure below). The number of patients in both groups is now reported in Tables.

**Figure**. Distribution of diagnosis time.

**2. Please add references for the definition of iTTP.**

The reference defining iTTP ( “Scully M, Cataland S, Coppo P, et al. Consensus on the standardization of terminology in thrombotic thrombocytopenic purpura and related thrombotic microangiopathies. *J Thromb Haemost*. 2017;15(2):312‑322.) is cited at position 27, we now refer to it earlier, thus it now appears in position 2.

We also deleted the first reference “Shatzel JJ, Taylor JA. Syndromes of Thrombotic Microangiopathy. *Med Clin North Am*. 2017;101(2):395‑ 415” and replaced it by “Kremer Hovinga JA, Coppo P, Lämmle B, Moake JL, Miyata T, Vanhoorelbeke K. Thrombotic thrombocytopenic purpura. *Nat Rev Dis Primer.* 2017;3(2):17020.”

**3. Please add details on the treatment of iTTP.**

We moved the Table S1 “Comparison of other outcomes” from the supporting information to the manuscript as Table 3. It contains information regarding treatment in both groups. We changed its title for “Comparison of secondary outcomes”.

**4. How to define “brain dysfunction” and “brain dysfunction free survival”? and related references should be provided.**

Neurological symptoms in TTP include headache, altered mental status, transient ischemic attack or stroke, and seizure. We used the term “brain dysfunction”, in order to remind the reader that isolated headaches were not included in this definition, as mentioned in the method section.

We acknowledge that this term is not straightforward. We now use the term “Neurological event” instead in the text, tables and figures.

**5. The authors did not observe the effect of diagnostic delay on mortality. Why?**

As mentioned in the “Results” section, only 2 patients died, from disease onset to hospital discharge (ie. mortality = 5%). One patient died in each group; therefore, mortality did not differ between the immediate and delayed diagnosis groups. Our study is underpowered to detect an impact on mortality, which would probably require studying hundreds or even thousands of cases. Also, importantly, we could have missed some undiagnosed iTTP patients with a fatal outcome.

**Reviewer #2:** The current article from Renaud and colleagues investigates the effect and reasonings of diagnostic delay of TTP and neurological outcomes in affected patients. Outlining the diagnostic during the differential diagnosis between TTP and other diseases is of great interest; however, several significant questions are raised. Moreover, the manuscript at this stage provides only confirmatory results that >24h delay markedly increase negative consequences. The delay in diagnosis directly correlates with the delay in a specific treatment, and the consequences of the latter are reported by Sawler et al., 2020

*Major comments:*

**1. Although the diagnosis establishment is essential, how does it correlate with the plasma exchange therapy start? Are there patients from the group with the immediate diagnosis but delayed plasma transfusions?**

The patients included in our study were taken care of in France, at University Hospital of Nantes, or in the regional surrounding hospitals. Our county (44, Loire Atlantique) is small (6 880 km², 1,426 million inhabitants), thus plasma exchanges are quickly accessible and are routinely initiated within hours, and in any case less than 24h. If PE is not quickly available (night), our practice is to initiate plasma infusions. Accordingly, no timely diagnosed patients were found to receive plasma therapy more than 24h after admission.

**2. How was the cut-off for the delayed and immediate diagnosis determined?**

As there is no significant difficulties with access to plasma exchange, and given that the exact hour of a definite diagnosis is not mentioned routinely in clinical notes, we used therapy initiation (plasma exchange or plasma infusion) as a marker of diagnosis.

We chose this 24h cut-off because we considered that it best reflected misdiagnosis. A shorter cut-off would capture both logistical issues and misdiagnosis, whereas a longer cut-off wouldn’t be in accordance with expert recommendation regarding TTP management. Another reason was the distribution of data (Figure below). It allowed us to confirm the cut-off of 24h to differentiate patients with delayed or immediate diagnosis

**Figure**. Distribution of diagnosis time.

**3. In what number of patients were the ADAMTS13 levels determined before or shortly after the plasma exchange therapy?**

Unfortunately, we don’t have access to this specific information. Usually, sample for ADAMTS13 activity was realized before plasma therapy initiation, and results were not returned until at least 48-72h. All patients had an ADAMTS13 activity level < 5%, as mentioned in the methods section.

**4. The authors state that different therapeutic approaches were used. Please, specify what treatment patients received in different groups and their counts.**

These data were initially reported in the supporting information Table S1 untitled “Comparison of other outcomes”. We now include it in result section of the manuscript, as Table 3, with the title “Comparison of secondary outcomes”

**5. Several prediction scores were developed to assess TTP risks. Did clinicians use them to evaluate the probability of TTP? It is interesting to check if they are significantly different between derived groups.**

TTP prediction scores, especially PLASMIC and French scores, are used by specialized clinicians in our center, when TMA is suspected. However, they are not used by non-specialized first line practitioners. Indeed, as we have shown, a significant proportion of clinicians, at the very first hospital evaluation, do not suspect the TMA but rather immune thrombocytopenic purpura (ITP). TTP prediction scores will be added to our local recommendations regarding the evaluation of patients with suspected ITP / unexplained thrombocytopenia.

We agree that the retrospective calculation of prediction score based on data available at first evaluation can be informative. Thus, we added the French score at first hospital visit in the Table 1. The score was ≥1 in all cases, and reached 2 for 19/20 patients (95%) in the immediate diagnosis group and 14/18 patients (78%) in the delayed diagnosis group (p=0.17). This result highlights the fact that TTP probability was high in both groups without significant differences. Information on the French score and the corresponding reference have been added in the “Method” section.

Unfortunately, the PLASMIC score’s determination requires INR and hemolysis work up. Missing data preclude its calculation at first hospital visit in many patients, especially in the delayed diagnosis group. Calculating it using follow-up data would be inaccurate (risk of overestimation).

**6. The Clinical-biological determinants of diagnostic delay section raise several comments:**

**a. Although the authors state that the group has statistically different PLT counts, one would argue that both levels might be considered as severe thrombocytopenia.**

We totally agree that this platelet count difference between the two groups is not clinically relevant. We believe that it reflects a less advanced microangiopathy but it is unlikely to contribute to clinicians misreasoning (unlike hemoglobin levels). We therefore deleted this information from the text of the “Results” section. These data and statistics remain in Table 1.

**b. Mixing values from total counts and delayed group when analyzing schistocytes strongly confuses the reader**

We acknowledge that moving from group comparison to a global analysis of schistocytes data may be a bit confusing. However, we believe that it demonstrates the impact of a low schistocytes count on clinical reasoning and patients’ management.

We now remind that we refer here to the very first hospital visit. We added the mention “at first hospital visit” near “schistocytes” in this section of the “Results” part for more clarity.

*Minor comments:*

**1. Please, specify the ADAMTS13 assay type used in the study.**

The ADAMTS13 activity was determined by a method using commercial recombinant FRETS-VWF73 peptide, and anti-ADAMTS13 autoantibodies were screened using a commercial ELISA kit. All ADAMTS13 assays were performed in Agnes Veyradier’s Hematology Laboratory at Lariboisière Hospital, AP-HP, Paris.

**2. Table 1 and Table 2 content is not immediately straightforward for the reader. Two subcolumns are reported in each section; however, it’s not apparent what is reported in each one and in what units. For example, Women 26 (68). I strongly recommend reformatting the tables to include all the numbers**

As suggested, we reformatted the Table 1 and 2, as well as the Table 3 (added from supporting information). We now report all units, proportions (%), interquartile range (IQR), and minimum-maximum (range). We deleted the footnote “^a^”, and changed the title for “Comparison of neurological outcomes from disease onset to hospital discharge” for the Table 2.

**3. The manuscript requires proof-reading by an English native person**

As required, an English native person read and edited the manuscript where needed.

**Journal Requirements**

**1. Please ensure that your manuscript meets PLOS ONE's style requirements, including those for file naming.**

We edited our manuscript according to PLOS ONE’s style requirements.

**2. Thank you for your submission, and your ethics statement reading, "This study is in accordance with the Declaration of Helsinki, and the French Data Protection Authority and Legislation (MR003 reference methodology). No change in the current clinical practice and no randomization were performed. As it was a retrospective study, according to the French legislation (articles L.1121-1 paragraph 1 and R1121-2, Public health code), approval of the ethics committee was not needed to use data for this study."**

**At this time, we request that you please indicate whether all data was anonymized and/or aggregated prior to your access and analysis, or whether authors had access to identifying patient information. Thank you for your attention to this request.**

We added the sentence “Data were manually extracted from medical charts (AR and AC), containing patient identifying information, and anonymized before storage and data analysis.” In the Data collection subsection.
