# Supplementary material for: Deleterious neurological impact of diagnostic delay in immune-mediated thrombotic thrombocytopenic purpura
Source: PLoS One. 2021 Nov 19;16(11):e0260196. doi: 10.1371/journal.pone.0260196 (PMC8604371; doi:10.1371/journal.pone.0260196)
Supplement: Attachment — Submitted filename: Response to reviewer.docx [file pone.0260196.s003.docx]

**Response to reviewer**

**Reviewer #3:**

**ABSTRACT

> Unexpectedly, recent studies suggest that a slight delay in TPE initiation has no significant impact on patients’ outcome.**

**Some other expressions like "****However, the exact impact of a slight delay in TPE initiation on the subsequent patients’ outcome is still controversial” would be better.**

We agree that this formulation is more relevant. We changed this sentence as suggested.

**> delayed (>24h, group 1) and immediate diagnosis (≤24h, group 2).

24 hours from when? From the clinical onset, or from the first hospital visit?**

The 24 hours cut-off refers to the time from first hospital visit to therapeutic plasma exchange (TPE) initiation. We specified this information in the abstract.

**> Conclusion: Diagnostic delay is highly prevalent in iTTP, with a significant impact on neurological outcome.

The shown results imply that the occurrence of stroke/TIA during hospitalization may be slightly higher in “delayed” group, whereas the occurrence of irreversible neurological sequelae was not significantly different (p=0.22) between the groups. Thus, this conclusion is misleading. The swift performance of TPE may suppress the occurrence of stroke/TIA, but it may not improve the neurological outcome.**

We agree that if one only considers long term / irreversible neurological sequelae as the neurological outcome, this part of the abstract’s conclusion is misleading. We therefore modified it for “Diagnostic delay is highly prevalent in iTTP, with a significant impact on short-term neurological outcome”.

**> Interdisciplinary efforts are necessary in order to increase first-line physicians’ awareness regarding the early clinical-biological presentation of iTTP.

What is the author’s specific message regarding the suggested factors of delayed diagnosis? Please describe more specifically. Is there a supporting data to say that the “unawareness” among the first-line physicians of the disease concept of “TTP” caused the delayed diagnoses in your cohort?**

We agree that this formulation is vague. In our opinion, the hypothesis of the “unawareness” of the frontline physicians is clearly supported by our finding that incomplete or missing hemolysis work-up was significantly more frequent in the delayed diagnosis group, which means that thrombotic microangiopathy was not suspected initially. This “unawareness” also concerns the concept of “TTP” regarding the interpretation of schistocytes. Indeed, schistocytes were significantly more frequently <1% in the delayed diagnosis group, when searched, and consider negative. We therefore modified this sentence for “In patients with profound thrombocytopenia, the thorough search for signs of incipient organ dysfunction, systematic hemolysis workup, and proper interpretation of schistocytes count are the key elements of early diagnosis of TTP.”

**METHODS

Graph Pad Prism software 6.0 >> GraphPad Prism software 6.0
Please add the information about the manufacturer of GraphPad Software.
e.g., (GraphPad Software, Inc., San Diego, CA)**

We did the modification and added the information about the manufacturer of GraphPad Software as requested.

**I cannot find the criteria and rationale to divide the cohort to the “delayed” group (>24h) and “immediate” group (<24h) in the METHODS section. From when the “24 hours” was counted? From the clinical onset? Why the cutoff time of 24 hours was set? Did previous studies support the cutoff at 24 hours of TPE initiation from onset to influence the clinical outcome?**

As specified in the “Data collection” part of the “Methods” section, the diagnosis was considered delayed when TPE was initiated more than 24 hours after the first hospital visit

In our county (44, Loire Atlantique, 6 880 km², 1,426 million inhabitants), access to plasma therapy (TPE or plasma infusions) is always possible within 24hours. Indeed, if TPE is not quickly available (night), our practice is to initiate plasma infusions first. Accordingly, no timely diagnosed patients were found to receive plasma therapy more than 24h after admission. We chose this 24h cut-off because we considered that it best reflected misdiagnosis. A shorter cut-off would capture both logistical issues and misdiagnosis, whereas a longer cut-off wouldn’t be in accordance with expert recommendation regarding TTP management. Another reason was the distribution of data (Figure below).

**Figure**. Distribution of diagnosis time.

In the study of Sawler et al. (reference 11 in the manuscript), the cut-off of 24hours for PE initiation delay have also been used (along with 8hours for a first level of analysis). It was found in 24% of acute episode of iTTP (first or relapse) and it tended to be associated with a higher risk of thrombotic event (aHR=2.9, 95% CI=0.6-12.8) and death (aHR=1.40, 95% CI=0.20-9.79) that did not reach statistical significance.

**RESULTS

> Clinical- biological determinants of diagnostic delay

How about performing a multiple regression analysis by using the required days from onset to TPE initiation as the dependent variable? Currently, the authors only performed univariate analyses to search for the determinants of diagnostic delay.**

As most clinicians we consider that TTP should be diagnosed clinically at first presentation, and TPE initiated pending ADAMTS13 activity results. This is why we chose to study patients with vs without delayed diagnosis, i.e as a categorical variable, but not “diagnostic delay” as a quantitative variable. We feel that 2 weeks versus 4 days of diagnostic delay does not really inform us on physician clinical reasoning at first hospital presentation. However, it is true that a larger patient population (multicenter) may allow to identify additional factors that could explain why some patients with delayed diagnosis experience a longer diagnostic delay than others.

As for the absence of multivariate analysis (e.g. logistic regression between immediate and delayed diagnosis) we believe that it would have limited relevance: given our limited statistical power it may overemphasis on variable with the highest statistical significance but not the highest clinical relevance.

For example, platelet count is significantly higher in patients with delayed diagnosis, but still very low, and it is unlikely to contribute to misdiagnosis (unlike the absence of anemia).

**TABLES

Table 1:

How were the data regarding the prevalence of fever at the first hospital visit in the two groups?**

Fever was present in 5/18 patients (28%) of the delayed diagnosis group and in 2/20 patients (10%) in the immediate diagnosis group (p=0.22). We added 2 lines in the Table 1 below “Constitutional signs” to specify the prevalence of “Fever” and “Fatigue”.

**> no. (%)

What is “no”? Maybe, “n” would be better, if the authors intended to show the numbers.**

“no.” corresponds to the numbers. We changed it for “n” in all the tables as suggested.

**> points (IQR)**

**Are the shown values median or mean?**

The shown values are median as specified in the “Statistical analysis” part of the “Methods” section.

**(N=38) (N=20) (N=18) >> (n=38) (n=20) (n=18)
“N” and “n” have different meanings.**

We did the modification as suggested

**Table 2:

> Table 2. Comparison of neurological outcomes from disease onset to hospital discharge

I think the table should compare the neurological outcomes from “TPE initiation” to hospital discharge, because this section intends to show the “Deleterious neurological impact of diagnostic delay”. If they include the time “from disease onset” in this table, the numbers overlap with those in Table 1.**

According to us, comparing only neurological events from TPE initiation to hospital discharge would ignore an important information regarding neurological events occurring between first hospital visit and TPE initiation (median=5 days in the delayed diagnosis group).

The purpose of the Table 2 is to focus on short-term neurological outcomes, encompassing the patient’ history from disease onset to hospital discharge, and to highlight the repercussion of delaying TPE initiation. By contrast, Table 1 data only refers to baseline data. It is true that for the handful of patients who presented a neurological event before the hospital visit but none afterwards, this will appear in both tables, but we believe that this is the only way to describe patients’ symptoms and short-term outcome.

**> a Number of neurological events [number of patients].

Which of the “number of events” or “number of patients” was used to calculate the p-values by performing chi square or Fisher test?**

We used the number of patients to calculate the p-values by performing a Fisher’s exact test. We specified this information in the footnote of the Table 2.

**> Stroke/TIA [n] a

Please separately show the numbers for “stroke” and “TIA”. Did skilled neurologists diagnosed the TIA conditions? Were all episodes of stroke confirmed by brain MRI scan?**

In our center (University Hospital of Nantes, Hotel-Dieu, France), the access to a neurologist’s evaluation is not possible 24hours/24 7days/7. So, not all patients with TIA conditions have been diagnosed by a “skilled neurologist”. Of note, 50% of strokes or TIA not related to TTP are outside neurology department. Thus, general internist or intensivist who manage these patients have sufficient experience to identify TIA/strokes.

Unfortunately, access to brain MRI scan is limited in France, even for patients who suffer an unexplained stroke. This is a matter of intense debate for decades. French physicians regularly mention that there is less MRI scan per inhabitant in France than in other countries (such as Turkey for example). All patients had a brain CT scan which excluded intracranial hemorrhage. Only 1 patient had a brain MRI. Of note, we fell that requesting a brain MRI is not a priority in TTP patients with a normal CT scan: it may delay therapeutic management (transfer to the ICU, TPE initiation) and it is even more time consuming in ICU patients. As the current definition of TIA relies on the lack of brain lesion on MRI, we fell that it would be incorrect to report TIA and strokes separately (which were recorded as a unique data). In our view the most important point is whether a patient died or suffered permanent deficits, which is reported in table 2.

**Table 3:

> TPE per patient – no. (IQR)b
Maybe, TPE “cycles” per patient?**

We added “cycle” to TPE in Table 3 as suggested.

**Journal requirement**

**Please review your reference list to ensure that it is complete and correct. If you have cited papers that have been retracted, please include the rationale for doing so in the manuscript text, or remove these references and replace them with relevant current references. Any changes to the reference list should be mentioned in the rebuttal letter that accompanies your revised manuscript. If you need to cite a retracted article, indicate the article’s retracted status in the References list and also include a citation and full reference for the retraction notice.**

We made sure that the references were complete and correct. We did not add or delete any reference.
